# Human monoclonal antibodies isolated from a primary pneumococcal conjugate Vaccinee demonstrates the expansion of an antigen-driven Hypermutated memory B cell response

**DOI:** 10.1186/s12879-018-3517-7

**Published:** 2018-12-04

**Authors:** Zhifeng Chen, Kara S. Cox, Aimin Tang, Jeanette Roman, Malorie Fink, Robin M. Kaufhold, Liming Guan, Andy Xie, Melissa A. Boddicker, Debra Mcguinness, Xiao Xiao, Hualin Li, Julie M. Skinner, Thorsten Verch, Mary Retzlaff, Kalpit A. Vora

**Affiliations:** 1 Department of Infectious Diseases and Vaccines Research, Merck & Co., Inc, Kenilworth, NJ USA; 2 Department of Vaccine Analytical Development - Immunoassay, Merck & Co., Inc, Kenilworth, NJ USA

**Keywords:** Monoclonal antibodies, Human, Plasmablast B cell, Pneumococcal conjugate vaccine

## Abstract

**Background:**

Community-acquired pneumonia is a leading infectious cause of hospitalization. A few vaccines exist to prevent pneumococcal disease in adults, including a pneumococcal polysaccharide unconjugated vaccine and a protein conjugated polysaccharide vaccine. Previous studies on the human immune response to the unconjugated vaccine showed that the vaccine boosted the existing memory B cells. In the present study, we investigated the human B cell immune response following pneumococcal polysaccharide conjugate vaccination.

**Methods:**

Plasmablast B cells from a pneumococcal polysaccharide conjugate vaccinee were isolated and cloned for analysis. In response to primary vaccination, identical sequences from the plasmablast-derived antibodies were identified from multiple B cells, demonstrating evidence of clonal expansion. We evaluated the binding specificity of these human monoclonal antibodies in immunoassays, and tested there in vitro function in a multiplexed opsonophagocytic assay (MOPA). To characterize the plasmablast B cell response to the pneumococcal conjugated vaccine, the germline usage and the variable region somatic hypermutations on these antibodies were analyzed. Furthermore, a serotype 4 polysaccharide-specific antibody was tested in an animal challenge study to explore the in vivo functional activity.

**Results:**

The data suggests that the pneumococcal polysaccharide conjugate vaccine boosted memory B cell responses, likely derived from previous pneumococcal exposure. The majority of the plasmablast-derived antibodies contained higher numbers of variable region somatic hypermutations and evidence for selection, as demonstrated by replacement to silent ratio’s (R/S) greater than 2.9 in the complementarity-determining regions (CDRs). In addition, we found that VH3/JH4 was the predominant germline sequence used in these polysaccharide-specific B cells. All of the tested antibodies demonstrated narrow polysaccharide specificity in ELISA binding, and demonstrated functional opsonophagocytic killing (OPK) activity in the MOPA assay. The in-vivo animal challenge study showed that the tested serotype 4 polysaccharide-specific antibody demonstrated a potent protective effect when administered prior to bacterial challenge.

**Conclusions:**

The findings on the pneumococcal polysaccharide conjugate vaccine responses from a vaccinated subject reported in this study are similar to previously published data on the pneumococcal polysaccharide unconjugated vaccine responses. In both vaccine regimens, the pre-existing human memory B cells were expanded after vaccination with preferential use of the germline VH3/JH4 genes.

**Electronic supplementary material:**

The online version of this article (10.1186/s12879-018-3517-7) contains supplementary material, which is available to authorized users.

## Background

*Streptococcus pneumoniae* (also called pneumococcus) is a gram-positive bacterium that usually shows as a diplococcus or short chains of cells. It was first isolated by Pasteur and Sternberg in 1881 and is the most frequent cause of lower respiratory tract infection [1]. Community-acquired pneumonia is a leading infectious cause of hospitalization, the annual incidence of Pneumococcal pneumonia is 24.8 cases per 10,000 adults in USA reported from a large scale survey from 2010 to 2012 [2]. Annually, over 1 million infants and adults die of *S. pneumoniae* - related diseases globally [3]. Pneumococcal pneumonia is a common lethal secondary infection of influenza. More than half of the people who died in the 1918 influenza epidemic (causing 50–100 million death toll) died of invasive pneumococcal disease [4].

There are over 90 different serotypes of *S. pneumoniae* grouped by the composition of their polysaccharide capsules [5–7], and the polysaccharide capsule is the most important virulence determinant for pneumococci. It is critical in colonization, invasion and dissemination from the respiratory tract [1]. The risk of invasive disease depends on serotype; some serotypes are benign, whereas other serotypes can lead to invasive disease. A pneumococcal polysaccharide vaccine (Pneumovax®23, or PPSV23) was licensed in 1983. It contains polysaccharide antigens from 23 serotypes of pneumococcal bacteria that in total cause approximately 80–90% of bacteremic pneumococcal disease. The PPSV23 formulation contains the following capsular serotypes: 1, 2, 3, 4, 5, 6B, 7F, 8, 9 N, 9 V, 10A, 11A, 12F, 14, 15B, 17F, 18C, 19F, 19A, 20, 22F, 23F, and 33F [7]. This vaccine can induce T-cell-independent B cell responses. However, this vaccine didn’t show efficacy in infants, a population which lacks T-cell-independent responses which are required to generate antibodies against long-chain polysaccharides [1].

A 7-valent pneumococcal polysaccharide conjugate vaccine (Prevnar 7®, or PCV7) was introduced in 2000. It includes purified capsular polysaccharides of seven serotypes of *S. pneumoniae* (4, 6B, 9 V, 14, 18C, 19F, 23F) conjugated to a nontoxic variant of diphtheria toxin known as CRM197 [8]. In 2010, a 13-valent pneumococcal conjugate vaccine (Prevnar 13®, or PCV13) was licensed in the United States which contains the seven serotypes of PCV7 plus serotypes 1, 3, 5, 6A, 7F and 19A also conjugated to CRM197. PCV13 can induce T-dependent B cell responses, and showed efficacy in protecting both adults and infants from pneumococcal infections [7, 9, 10].

Cloning of monoclonal antibodies from human B cells has been very successful during the past decade, and this technology has enabled the study of human B cell responses following vaccination or natural infection [11–19]. In a study reported by Smith K et al., PPSV23 can induce a highly mutated and anamnestic B cell response against pneumococcal polysaccharide antigens in previously experienced donors during primary vaccination [15]. In this study, we collected blood from a PCV13 vaccinee 9 days post vaccination to better define the humoral immune response to a pneumococcal conjugate vaccine. Here, we show that the majority of the plasmablast-derived antibodies contained a high variable region hypermutation frequency, which suggests that these antibodies were elicited from existing memory B cells in this donor. This data suggests that the PCV13 expands the memory B cell pool derived from previous polysaccharide exposure. Together, these findings show PCV13 and PPSV23 both can boost previously existing memory B cells against pneumococcal polysaccharides.

Furthermore, isolated polysaccharide specific antibodies demonstrated in vitro OPK activities and one of the mAb tested was able to protect mice in a passive challenge model in mice. These results suggest that the antibodies have the potential for therapeutic purposes for the prevention or treatment of pneumococcal associated diseases.

## Methods

### Human subject and PBMC preparation

Blood samples were obtained with informed consent from a donor who was vaccinated with Prevnar13®. Peripheral blood mononuclear cells (PBMC) were purified from blood collected in EDTA tubes by density gradient centrifugation in histopaque over Accuspin™ tubes (Sigma Aldrich) according to the manufacturer’s instructions. Blood was collected nine days after vaccination for the plasmablast isolation and used fresh.

### Single human plasmablast B cells sorting

Fresh PBMC were used to sort human plasmablast B cells as described before [12]. Cells were stained with the following antibody panel: CD3 BV421 (BD Biosciences), CD19 APC (BD Biosciences), CD27 FITC (BD Biosciences), CD38 PE Cy7 (BD Biosciences), CD20 PE (BD Biosciences). Plasmablasts were defined as CD3^−^/CD19^+^/CD27^+^/ CD38^+^/CD20^−^ and sorted on a FACS Jazz sorter in single cell mode into a 96 well plate.

### PCR amplification of antibody genes

Single plasmablast cells were sorted into 96-well PCR plates (Bio-Rad) containing RNase inhibitor RNasin® (Promega) in each well [12]. Qiagen One-step RT-PCR kit (Qiagen) was used in RT-PCR to amplify antibody heavy and light chain genes.

The RT-PCR products were used directly as templates in nested-PCR to amplify antibody variable regions with Invitrogen pfx50 DNA polymerase (Invitrogen), Detailed methods were published previously [19].

### Production of recombinant antibody in mammalian cells

The discovered antibody sequences were applied to codon optimization and gene synthesis; the synthesized DNAs were cloned into pTT5 human IgG1 expression vector for mammalian cell expression. Detailed procedure was described before [20].

#### Polysaccharide-specific Sandwich ELISA

A two-day polysaccharide-specific sandwich ELISA was used to identify polysaccharide-binding mAbs.

Day one: An anti-polysaccharide mouse mAb (MSD proprietary) for capture were coated to plate at 1 μg/mL. Mouse antibody coated plates were washed, blocked, and then incubated overnight at 2–8 °C with serially diluted multivalent polysaccharides (Pneumovax23 positive control without adjuvant, MSD).

Day two: Recombinant human mAbs were diluted to 1 μg/mL and applied to plates for 1 h. Plates were washed, then incubated for one hour with 1:6000 dilution of donkey anti-human antibody conjugated with alkaline phosphatase (Jackson ImmunoResearch) in a solution of assay diluent spiked with normal mouse serum (Jackson ImmunoResearch). Plates were washed and 4-MuP substrate (Virolabs) was applied for 45 min, absorbance was read at 360 nm (excitation)/ 450 nm (emission) on a SpectraMax M2E spectrophotometer.

Raw data were collected using SoftMax Pro (V5.4, Molecular Devices), exported into Excel, and then analyzed with Prism (Graphpad).

### MOPA (multiplexed Opsonophagocytic assay) for functional polysaccharide-specific monoclonal antibodies

Pneumococcal multiplexed opsonophagocytosis assays (MOPA) were performed as described previously [21, 22] . Following the incubation of mixture of antibody, bacteria, complement (Pel-Freez Biologicals) and HL-60 cell (ATCC), 10 μl reaction was transferred to an individual well on a Costar (Corning) or Millipore 96-well filter plate, 100 μl THYE broth (Todd Hewitt Yeast Extract medium, Teknova) was added to each well. The plates were placed in sealed plastic bags and incubated overnight at 37 °C (serotype 3 bacteria plate was incubated at 27 °C). Plate filters were stained with 100 μl/well of a 0.025% Coomassie blue solution (0.1% Coomassie blue for serotype 3) (Bio-Rad). Colonies were destained with Coomassie destaining solution (Bio-Rad) and vacuum filtered until dry. Stained bacterial colonies were counted on a CTL Immunospot reader. The OPK (OpsonoPhagocytic Killing) titers were calculated as the reciprocal of the antibody dilution with 50% killing compared to the average growth in the complement control (no antibody control) wells using the Opsititer 3 software licensed from UAB Research Foundation.

### In-vivo evaluation of polysaccharide-specific monoclonal antibody protection from S. pneumoniae serotype 4 challenge

All animal experiments were approved by the Institutional Animal Care and Use Committee (IACUC), Merck & Co., Inc. (Kenilworth, NJ, USA). All procedures were performed in accordance with our institution’s IACUC guidelines in strict accordance with the recommendations in the Guide for Care and Use of Laboratory Animals of the National Institutes of Health.

Female C57BL/6 mice from Charles River Laboratories (Wilmington, MA) were housed in large mouse containers (*n* = 5 mice/box) with microisolator lids, and the rooms were maintained with controlled humidity and temperature, and 12 h light-dark cycles. All containers had nestlets and animals were provided standard chow (Purina 5001 rodent diet) and water ad libitum. The physical condition of the animals was monitored daily, and any health changes were noted.

For humane reasons, any animal identified as moribund and unable to move about or access food and water throughout the course of the study were sacrificed using CO_2_ inhalation (10–30% CO_2_ in the air mixture inhaled per minute); upon loss of consciousness, the animals were euthanized by cervical dislocation. At the end of the study, all remaining animals were euthanized using CO_2_ inhalation followed by cervical dislocation.

### Passive transfer of monoclonal antibodies

Monoclonal antibodies, *S. pneumoniae* serotype 4 specific 1A10 or isotype control *S. pneumoniae* serotype 6 A/B specific 1C4, were diluted in saline to the appropriate working stock dilution (0.2, 0.02 or 0.002 mg/ml). Diluted monoclonal antibodies (100 μl) were administered intravenously via the tail vein approximately 24 h prior to bacterial challenge.

#### Bacteria

*S. pneumoniae* serotype 4 strain TIGR4 [23] (obtained from Dr. Carlos L. Orihuela, University of Alabama at Birmingham) was grown on tryptic soy agar with 5% Sheep’s Blood plates (blood agar plates; Remel) overnight and in Todd Hewitt Broth (Teknova) at 37 °C in 5% CO_2_ for working cultures. Pneumococci at exponential phase of growth, optical density (OD)_620_ = 0.2, were used for the infection.

#### Infection

The challenge dose was a sublethal dose that targeted 20% survival of naïve mice, determined prior to intiation of the current experiments.. Bacteria were grown as described above and inoculum was prepared by diluting the bacteria with sterile phosphate-buffered saline (PBS) to the final desired concentration (~ 1 × 10^6^ CFU/ml). The number of CFUs used for challenge was confirmed after infection by serial dilution of the inoculum, plating on tryptic soy blood agar plates, overnight incubation, and extrapolation from colony counts. Oropharyngeal aspiration in 14 week old mice was performed following the protocol described by González-Juarbe et al. [24]. Briefly, mice were anesthetized with 2.5% vaporized isoflurane (Abbott Laboratories) and placed upright by their incisors with their backs supported by a rodent intubation stand. The tongue was gently pulled outward using blunt forceps while pipetting 100 μl of the PBS bacterial suspension (~ 1 × 10^5^ CFU) into the oropharynx and accompanied by coverage of the nares with a finger until aspiration occurred. All mice recovered fully from anesthesia within 45 s of challenge.

#### Survival, weight change, and bacteremia

Infected mice were monitored for up to 7 days. The mice were monitored for clinical signs and survival twice daily for three days post-infection and then once daily through the end of the study. Weight loss was measured at 24, 48, and 72 h post-infection. For determination of bacteremia, 2ul of blood was collected via tail snips and transferred to 18ul of 300 USP Units/5 ml heparin (Sigma-Aldrich) in PBS at 24, 48, and 72 h post-infection. Serial dilutions of the blood were applied to blood agar plates and incubated overnight at 37 °C. Blood CFU values were extrapolated from colony counts.

## Results

### Isolation of pneumococcal polysaccharide specific antibodies from plasmablast B cells nine days post pneumococcal conjugate (PCV13) vaccination

In order to investigate the humoral immune response to PCV13, we collected whole blood from a vaccinated donor nine days after immunization. Freshly harvested peripheral blood mononuclear cells were isolated and stained with a panel of antibodies for the identification of plasmablasts, a population of antigen specific antibody secreting cells which peak in peripheral blood 5–10 days following vaccination. These cells were analyzed on a flow cytometric cell sorter by gating on the lymphocyte population, single cells, exclusion of CD3+ T cells, CD19 + and low cells, CD27 bright CD38 bright cells, and the exclusion of CD20 negative B cells (Additional file 1: Figure S1). A distinct population with the classical plasmablast phenotype CD27^HI^CD38^HI^ was observed, comprising approximately 0.88% of the B cells. (Additional file 1: Figure S1).

**Fig. 1:**
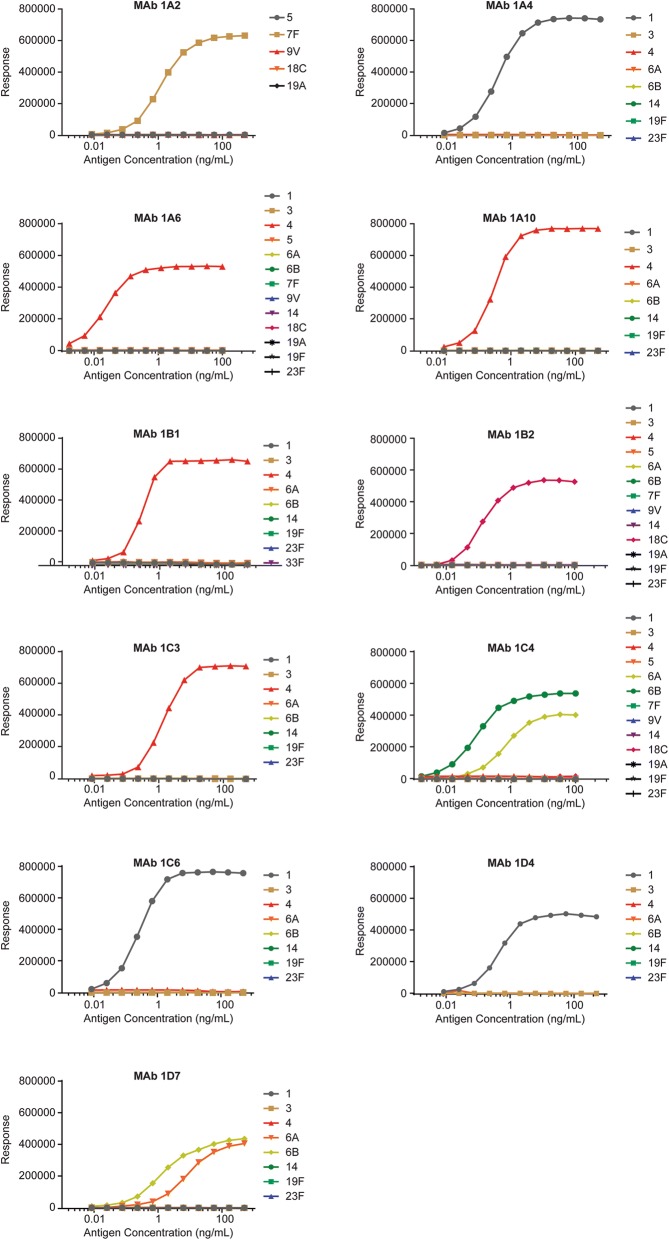
mAbs from PCV13-induced plasmablast B cells reactive to individual polysaccharide antigens. A two-day polysaccharide-specific sandwich ELISA was used to identify polysaccharide-binding mAbs . mAb 1A2 is reactive to polysaccharide type 7F. mAb 1A4, 1C6, 1D4 are reactive to polysaccharide type 1. mAb 1A6, 1A10, 1B4, 1C3 are reactive to polysaccharide type 4. mAb 1B2 is reactive to polysaccharide type 18C, and mAb 1C4, 1D7 are reactive to polysaccharide types 6A and 6B

Forty-eight of these defined plasmablast B cells were single sorted into a 96-well plate containing RNA extraction buffer. RNA was further subjected to RT-PCR and nested PCR procedures to amplify the variable region genes of the encoded immunoglobulins using primers as described previously [19]. Amplified heavy and light chains sequences obtained from single cell containing wells were recombinantly expressed as full length human IgG1 and tested in immunoassays for specificity against the individual polysaccharides of the PCV 13 vaccine by ELISA. Out of 35 wells with paired heavy and light chains, 18 of them showed binding to the polysaccharides of the vaccine. The remaining 17 antibodies did not bind to the antigens present in PCV13 and hence their specificity could not be determined. Several of the polysaccharide binding antibodies shared the heavy and light chains including junction regions suggesting repeated sampling of the individual clonally expanded sequences. Eleven polysaccharide-binding antibodies did not share their CDRs of heavy and light chains with other sequences, suggesting that they were unique antibodies (Table 1). Out of the 11 unique antibody sequences that bound polysaccharide antigens, mAb 1A6, 1A10, 1B4, 1C3 were reactive to serotype 4 polysaccharide, mAb 1A4, 1C6, 1D4 were reactive to serotype 1 polysaccharide, mAb 1C4, 1D7 were reactive to serotypes 6A and 6B polysaccharide, mAb 1A2 was reactive to serotype 7F polysaccharide, and mAb 1B2 was reactive to serotype 18C polysaccharide. The ELISA binding results are shown in Fig. 1. In this batch of 48 plasmablasts we did not detect antibodies binding to polysaccharide types 3, 5, 9 V, 14, 19A, 19F or 23F.

**Table 1:** Germline usage analysis for single plasmablast-derived antigen-specific mAbs

Antibody ID	Serotype(s)	VH			DH	JH	VL			JL
		Family	CDR3 Length	HCDR3 AA sequence	Family	Family	Family	CDR3 length	LCDR3 AA sequence	Family
1A4	1	3–23*04	10	DVRGSGSNSY	3–10*01	4*02	K2–30*01	9	MQGTYWPPIT	KJ2*01
1C6	1	3–53*01	8	EVDYAFDP	3–16*01	5*02	L8–61*01	10	VLFMGSGTWV	LJ3*02
1D4	1	3–74*03	8	SASGWYVN	6–19*01	4*02	K4–1*01	9	QHYASVPWT	KJ1*01
1A6 6 total	4	3–30-3*01	10	DPDTSNKIDY	2–2*02	4*02	K2–30*01	10	MQGTYWPPIT	KJ5*01
1A10	4	3–7*01	10	RMFGSSFRDY	6–6*01	4*02	L2–14*01	10	NSYTSSKTWV	LJ3*02
1B1	4	1–46*01	16	GGLLPGVAGATSPFQH	2–2*01	1*01	K3–20*01	9	QRYGSSPVT	KJ4*01
1C3	4	3–23*01	12	GPVLPAPKEFDY	2–15*01	4*02	L4–69*01	10	QTWDTVTNWV	LJ3*02
1C4 2 total	6A, 6B	3–7*03	8	EEWYRFDY	3–3*02	4*02	L2–8*01	10	SSHAGSKNVI	LJ2*01
1D7	6A, 6B	3–7*01	8	EIWFREDY	3–10*01	4*02	L2–8*01	10	GSRVGSNSVV	LJ2*01
1A2	7F	3–33*01	14	EPRAIADNYYGMDV	3–3*01	6*02	L10–54*01	11	SAWDSSLNAWV	LJ3*02
1B2 2 total	18C	3–7*01	9	LGGWRHLDY	3–16*02	4*02	K1–39*01	9	QQSYSSPYT	KJ2*01

To better understand the plasmablast B cell response to the pneumococcal conjugated vaccine, we analyzed the germline usage of the 11 unique antibodies (Table 1). CDRs and CDR3 length were determined with Kabat antibody V gene delineation system [25].

Germline usage of the mAbs was analyzed with IgBlast [26]. For heavy chain germline usage, the majority of the antigen-specific mAbs (94.44%, 17/18) used the VH3 germline, and 83.33% (15/18) used the JH4 germline, DH2, 3, 6 were frequently used. No germline usage preference was observed from the light chain genes, as kappa and light chain genes were evenly used. The heavy chain CDR3 length was between 8 and 16 (averaging 11.2) amino acids, and the light chain CDR3 length was between 9 and 11 (averaging 9.7) amino acids. Interestingly, we found four identical mAbs to polysaccharide type 4, two to polysaccharide type 18C, and two which dually recognized polysaccharide types 6A and B. The discovery of multiple clones with identical sequences demonstrates a clonal-expansion of B cells in this donor after vaccination. We further analyzed somatic hypermutation of variable regions of these antigen-specific mAbs, as higher amount of mutations are a hallmark of affinity matured B cells (Table 2).

**Table 2:** Analysis of replacement (R) and silent (S) mutations in heavy and light chain variable regions of single plasmablast-derived mAbs

Antibody ID	Number of clones	VH				VL			
		Total NT substitution (%)	R/S ratio in Frame work	R/S ratio in CDR regions	Strong Selection On HCDR	Total NT substitution (%)	R/S ratio in Frame work	R/S ratio in CDR regions	Strong Selection on LCDR
1A2	1	6.08%	4/2	10/2	Yes	0.34%	0/0	1/0	No
1A4	1	6.08%	3 /4	11/0	Yes	2.05%	1/0	4/1	Yes
1A6	6	8.59%	7/2	15/1	Yes	3.97%	2/1	8/1	Yes
1A10	1	6.46%	5/5	7/2	Yes	2.72%	0/1	7/0	Yes
1B1	1	1.36%	2/1	1/0	No	1.04%	0/0	3/0	No
1B2	2	4.42%	2/2	8/1	Yes	2.82%	3/0	4/0	Yes
1C3	1	5.78%	4/2	11/0	Yes	2.03%	1/1	4/0	Yes
1C4	2	5.48%	2/1	12/1	Yes	2.38%	5/2	0/0	No
1C6	1	3.75%	1 /2	8/0	Yes	2.37%	0/1	5/1	Yes
1D4	1	6.16%	5/4	9/0	Yes	3.64%	0/3	8/0	Yes
1D7	1	7.43%	5/3	14/0	Yes	2.74%	0/1	6/1	Yes

Note: Antigen-driven selection on HCDR and LCDR regions was judged based on somatic mutation analysis method developed by Shlomchik M et al. [27]

The antibodies listed in Table 2 had nucleotide (nt) substitution levels varying from 1.36% (4 nt) to as high as 8.59% (25 nt) in the heavy chain variable region, and 0.34% (1 nt) to 3.97% (12 nt) in the light chain variable region. Overall, heavy chain variable regions experienced more nucleotide substitutions than light chain variable regions. Shlomchik M, et al. [27] established a criterion for antigen-driven antibody somatic hypermutation and clonal selection. In this work, antibody variable domains, replacement (R) vs silent (S) ratio (R/S) greater than 2.9 in antibody CDR regions, and R/S greater than 1.5 in framework regions were considered a marker for heavy somatic hypermutation and antigen-driven selection. Among these 18 polysaccharide antigen-specific plasmablast B cells, 17 out of 18 (94.44%) showed strong selection (R/S > 2.9) in the Heavy chain CDR regions, and 14 of the 18 (77.78%) showed strong light chain CDR selections. As to the framework in antibody variable regions, 12 out of 18 (66.67%) in heavy chain and 10 out of 18 (55.56%) in light chain showed strong selection. These R/S ratio analysis results are in agreement with the nucleotide substitution analysis wherein both analysis showed heavy chain variable regions experienced more antigen-driven selection pressure than the light chain variable regions. We performed a similar analysis for memory B cell-derived mAbs following PCV13 immunization, and observed similar levels of nucleotide substitutions and R/S ratios in the memory B cell compartment (data not shown). This finding demonstrates that the majority of these B cells had experienced multiple rounds of affinity maturation in germinal centers, which indicates that the plasmablasts were expanded from memory B cells.

### Pneumococcal polysaccharide specific antibodies demonstrated functional in vitro opsonophagocytic killing (OPK) activities and in vivo protective effect

We picked 8 representative mAbs against the 6 serotype-specific polysaccharide antigens, and tested them in MOPA for in-vitro OPK activities (Table 3).

**Table 3:** OPK activities of mAbs (EC50, ng/ml)

		MOPA assay killing Pneumococcal serotypes															
Serotype	mAb	T4	T6b	T14	T23f	T18c	T19f	T9v	T6a	T1	T3	T5	T6c	T7F	T19A	T22F	T33F
T4	1A6	29	–	–	–	–	–	–	–	ND	ND	ND	ND	ND	ND	ND	ND
T4	1A10	55	–	–	–	–	–	–	–	ND	ND	ND	ND	ND	ND	ND	ND
T4	1B1	23	–	–	–	–	–	–	–	ND	ND	ND	ND	ND	ND	ND	ND
T18C	1B2	–	–		–	47	–	–	–	ND	ND	ND	ND	ND	ND	ND	ND
T1	1C6	ND	ND	ND	ND	ND	ND	ND	ND	14	–	–	–	–	–	–	–
T7F	1A2	ND	ND	ND	ND	ND	ND	ND	ND	–	–	–	–	11	–	–	–
T6a,b	1C4	–	37	–	–	–	–	–	100	–	–	–	142	–	–	–	–
T6a, b	1D7	–	355	–	–	–	–	–	334	–	–	–	2247	–	–	–	–

Note: - = no activity in MOPA assay (EC50 > 7500 ng/ml) [12]. ND = not tested. mAb serotype was determined by ELISA binding

Eight unique antibodies were chosen to assay for their in vitro OPK activity against their cognate Pneumococcal serotype strains. All antibodies had potent OPK activity with 50% effective concentrations (EC50’s) ranging from low ng/ml to μg/ml. Two mAbs (1C4 and 1D7) elicited OPK activity against closely related seorvariants 6A, B and C. Interestingly, 1C4 was 3X more potent on 6B than 6A and 6C. In contrast, 1D7 was equipotent on 6B and 6A but 6X less potent on 6C suggesting that 1C4 and 1D7 could be recognizing different epitopes on related polysaccharides. The remaining 6 antibodies (1A6, 1A10, 1B1, 1B2, 1C6 and 1A2) demonstrated antigen-specific OPK activities. Furthermore, there was no non-specific bacterial killing observed for any of the tested mAbs.

**Fig. 2:**
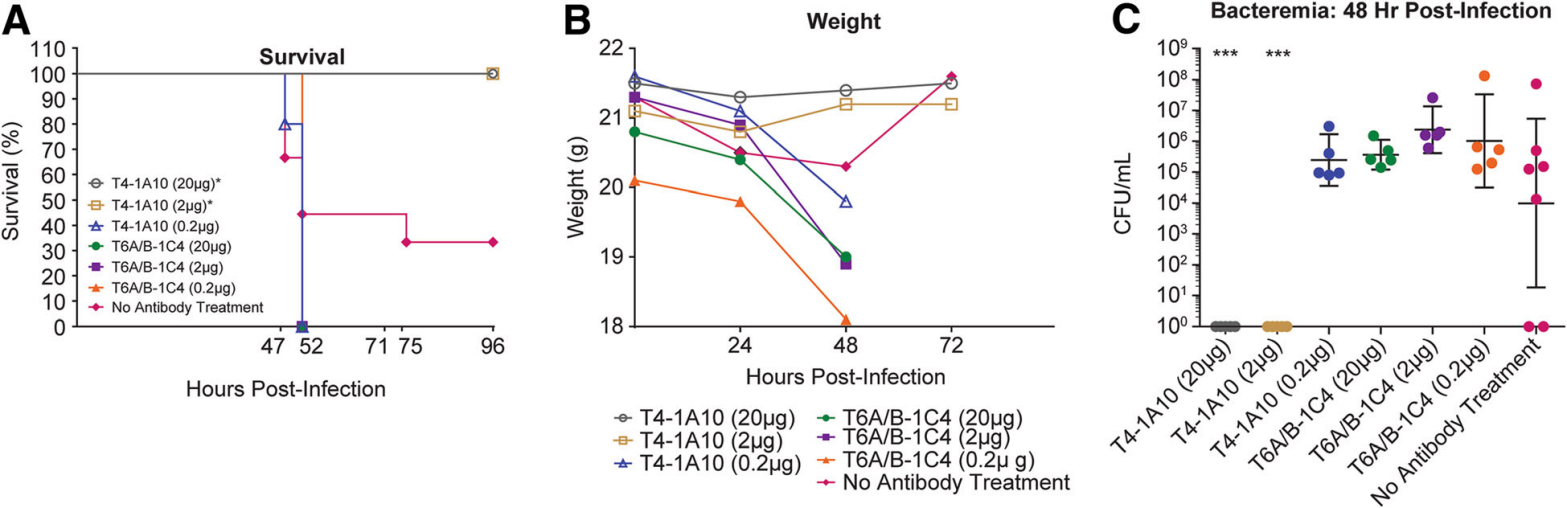
*S. pneumoniae* serotype 4-specific mAb protects mice from *S. pneumoniae* serotype 4 challenge. **a** Survival rate of each treatment group at 96 h post-infection. Log-rank (Mantel-Cox) test was performed. Each group was compared to No Antibody Treatment Group. * *p* < 0.05, statistically significant difference. **b** Mean weight of survivors in each group at 0, 24, 48, 72 h post-infection. **c** Bacteremia of each animal at 48 h post-infection. Individual raw data plotted on Log_10_ scale with geometric mean and 95% CI. Transformed data analyzed by One-way ANOVA with Dunnett post test to evaluate for significant reduction in blood CFU counts (compared to No Antibody Treatment Group), *** *p* < 0.001, highly statistically significant difference

To evaluate if the functional antibody activity observed in the in-vitro OPK assay could be translated into in vivo protection from infection, we further tested one of our PS serotype 4 specific antibodies, mAb 1A10, in a prophylactic murine challenge model. This antibody was selected as a representative antibody in this model due to the availability of the corresponding *S. pneumoniae* type 4 TIGR4 bacterial challenge mouse model in our laboratory. As an experimental control, an antibody produced with the identical IgG1 framework as the test antibody 1A10, but with specificity for *S. pneumoniae* serotype 6 A/B polysaccharide (mAb 1C4) was used as an isotype control. The serotype 4 specific antibody, 1A10, and the isotype control antibody were administered intravenously to five mice per group at 20, 2 or 0.2 μg per animal. Nine additional animals remained untreated as a comparator control group. 24 h after antibody dosing, *S. pneumoniae* serotype 4 strain TIGR4 was administered via oropharyngeal aspiration into each mouse. The mice were monitored for survival and weight loss after challenge, and blood was sampled to enumerate bacteremia (Fig. 2). All mice in the groups dosed with 20 or 2 μg per mouse of the T4 mAb 1A10 survived after 96 h (Fig. 2a). In contrast, mice dosed with the isotype control antibody 1C4, and the 0.2 μg dose of 1A10 died within 52 h of challenge (Fig. 2a). Weight loss was measured at 0, 24, 48 and 72 h post challenge as an indication of health for the surviving animals. The mice in the 20 μg and 2 μg groups of the T4 antibody 1A10 did not display weight loss after challenge (Fig. 2b). Although the mean weight loss rebounded for the no antibody control group, the weights represent the surviving mice (*n* = 3). The majority of mice in the no antibody control group did not survive the challenge by 72 h (Figs. 2a and b), however 3 out of 9 animals survived, likely due to the sublethal dosage. The target dose for challenge was determined using naïve mice to target 20% survival, while the no antibody control mice had 33% survival, which is in the acceptable range. To evaluate the level of bacteremia in the bloodstream, blood was sampled at 24, 48, and 72 h post infection for each surviving animal, diluted, and grown on blood agar plates. After an overnight incubation at 37 °C blood colony forming units were extrapolated from the colony counts. The bacteremia for each group at 48 h post infection is shown in Fig. 2c. In concordance with the complete protection from death and weight loss, the animals treated with 20 μg or 2 μg of the serotype 4 mAb 1A10 also did not have any level of blood bacteremia (Fig. 2c). Furthermore, animals with the lowest dose (0.2 μg) of 1A10 or the isotype control antibody at all doses showed as much or more bacteremia as the untreated animals, demonstrating a lack of protection (Fig. 2c). Overall, these results demonstrate that *S. pneumoniae* serotype 4 specific mAb 1A10, at 20 μg and 2 μg per animal can completely protect animals from challenge with a sub-lethal dose of *S. pneumoniae* serotype 4.

## Discussion

In order to understand humoral immune response elicited by the Pneumococcal conjugate vaccine we cloned individual vaccine-antigen specific antibodies from B cell plasmablasts post vaccination. Analysis of gene usage and mutational analysis of the immunoglobulin genes can divulge whether the responding plasmablasts are antigen experienced or derived from a de novo immune response. This information is particularly relevant in vaccines targeting the adult population, in which subjects could have had prior exposure to vaccine antigens. Boosting of memory B cell responses generally results in robust and affinity matured antibodies. Furthermore, this would also indicate that the vaccine antigen(s) are recognized by the immune cells in a similar manner as the native antigen present in the pathogen.

In the current study, we studied the human plasmablast B cell immune response from a pneumococcal conjugate vaccinee. Plasmablast B cells were collected 9 days after PCV13 vaccination from a single donor. A strong preference for usage of VH3 germline family (94.44%) and JH4 germline (83.33%) encoding antibodies was observed (Table 1). This observation is consistent with study reported by Smith K et al. [15] which showed that mAbs recovered from plasmablast B cells 7 days post-immunization with a PPSV23 also predominantly used the VH3 germline family (75.24%) and JH4 family (73.33%). Besides, the primer sets for RT-PCR and nested PCR had been extensively used in efficiently amplifying all major VH germlines [19], so primer bias is unlikely to be the reason for this high VH3 family germline usage. Moreover, this observation agreed with studies by Baxendale HE et al. in investigating pneumococcus specific human memory B cell repertoire [28]. Besides pneumococcal polysaccharide antigens, Adderson E et al. studied antibody VH usage in the human response to *Haemophilus influenzae* Type b capsular polysaccharide, and found all 15 mAbs identified were using VH3 germline [29]. Jia B et al. [30] recently published a study for comparison of B cell responses induced by PPSV23 and PCV13 in adult monkeys previously immunized with PCV7. Albeit there are some differences between immune responses induced by these two vaccines, this study showed these two vaccines induced similar scale polysaccharide-specific antibody secreting cells (ASCs, plasmablast B cells) at day 7 post boost-immunization, and there is no significant difference between polysaccharide- specific antibodies induced by the two vaccines in heavy chain gene-family usage 7 days after immunization. The researchers also pointed out that VH3 germline is the most often used while VH4 is also widely used, which may also be due to the differences in immune response of humans compared to non-human primates. These results from the non-human primate study as well as the data from other human responses to polysaccharides agree with our findings in humans of restricted germline VH usage. Considering around 35% of naïve B cells use the VH3 germline family [31], this polarized VH3 and JH4 germline usage suggests that the structure of VH3/JH4 antibodies may help them in binding to polysaccharide antigens. As an evidence, study by Bryson S et al. demonstrated that the structure of polysaccharide serotype 23F specific human antibodies (023.102 and pn132p2C05) using VH3/JH4 germline facilitates their binding to specific polysaccharide epitope by forming a deep crevice with the help of paired light chain. The crevice accommodates and engages the RGP epitope of 23F in several Van der Waals interactions and hence improves antibody binding to epitope [32]. Serotyope 4 mAb 1A6 is clearly elicited from an existing memory B cell clonal expansion, 6 out of the 18 cloned polysaccharide specific plasmablast B cells are sharing the same antibody heavy and light chain variable region genes as 1A6. mAb 1A6 has the same VH3–30/JH4*02 germline usage as mAb 023.102, and both of mAb 1A6 and mAb 023.102 are using kappa light chains, so 1A6 is chosen as representative for further in-silico modeling of antibody germline contribution to polysaccharide antigen binding. Using mAb 023.102 as the template, a model of mAb 1A6 also shows a crevice that accommodates a fragment of serotype 4 polysaccharide (Additional file 2: Figure S2). This further supports that VH3/JH4 germline usage may play a general role in forming crevice-like pockets for binding different polysaccharides.

To ascertain if the anti-polysaccharide antibodies isolated in the current studies were encoded by naïve or antigen specific memory B cells, we analyzed replacement (R) to silent (S) mutations in the heavy and light chain variable regions of plasmablast-derived mAbs. The mutation frequencies ranging from 1.36% (equivalent to 4 nt substitutions/V_H_ region) to 8.59% (25 nt substitutions/V_H_ region) suggest that most of these antibodies were encoded by antigen specific memory B-cells. The light chain variable region sustained considerable lower mutation frequencies (1 to 12 nt substitutions/V_L_ region) suggesting that the light chain may have less contribution towards binding of antigen. For the 18 polysaccharide antigen specific plasmablast B cells, further R/S ratio analysis found that the CDRs of VH chains had average ratios of 11.1 compared to an R/S average ratio of 2.1 in the framework, suggesting affinity maturation, a hallmark of memory B-cell encoded antibodies. Interestingly, the VL region had average R/S ratios of 10.4 and 1.8 in CDRs and FR respectively, again supporting the observations that the antibodies derived from plasmablast were encoded by memory B cells that had undergone affinity based selection during prior exposure to vaccine antigens. These frequencies of somatic mutations causing amino acid replacement predominantly in the CDRs match with data observed from anti-polysaccharide antibodies isolated from memory B-cells (data not shown). In addition, 9 out of 18 single sorted antigen-specific plasmablast B cells were serotype 4 specific, suggesting that the donor may have previously experienced serotype 4 capsular polysaccharide antigen. In total, this data suggests that the pneumococcal conjugate vaccine expands the existing memory B cell pool.

PPSV23 is an un-conjugated polysaccharide vaccine hypothesized to induce T-independent primary B-cell responses. In contrast, PCV13 is a protein-conjugate vaccine which would induce T-dependent primary B cell responses. Interestingly, both of these vaccines generated antibodies that were encoded by similar germline genes and contained comparable somatic hypermutations when given to adults. Smith K. et al. [15] analyzed mAbs generated from plasmablast B cells of four donors 7 days post-vaccination of PPSV23, and found that most of the mAb sequences were encoded by VH3 germline genes and contained somatic hypermutation with evidence for affinity based selection. In summary, the data reported here as well as Smith et al*’s* data suggested that both the conjugated and unconjugated pneumococcal polysaccharide vaccine boosts the pre-existing memory B cells generated during the primary exposure to the polysaccharide antigens most probably due to prior Pneumococcal exposure and colonization. It would be interesting to see a similar comparison of both vaccines in infants, which have lower exposure to the Pneumococcal strains. Interestingly, an adult NHP study evaluated the boosting of immune responses generated by both the unconjugated and conjugated vaccines, and found higher amplitude of the boost with conjugated vaccines with similar use of germline encoded antibodies between both groups [30].

All eight polysaccharide specific antibodies tested were functionally active as determined in an in vitro MOPA assay. Their specificities for opsonophagocytic activities were as predicted by ELISA. The antibodies demonstrated EC50’s from ng to μg/ml in the MOPA assay suggesting that they may play a role in vivo in preventing Pneumococcal infection. To test the in vivo functionality of the antibodies, we chose a representative monoclonal antibody 1A10 specifically for polysaccharide serotype4, and tested its’ ability to protect against the cognate Pneumococcal strain challenge in mice. Antibody 1A10 was selected for evaluation in the mouse challenge model due to its high binding to serotype 4 polysaccharide as well as the higher functional antibody titers (OPK activity) to serotype 4 *S. pneumoniae* bacteria. Amongst the three serotype 4 polysaccharide specific antibodies (1A6, 1A10, 1B1), 1A10 has OPK activity of IC50 = 55 ng/ml, which is slightly weaker than that of 1A6 (29 ng/ml) and 1B1 (23 ng/ml). Hence we believe that 1A6 and 1B1 will demonstrate in vivo activity based on their better OPK activities than 1A10 in vitro.. The antibody administered passively at doses as low as 0.1 mg/kg (mpk) dose was able to afford complete protection to animals challenged with Pneumococcal serotype 4. These results suggested that the polysaccharide-specific mAbs discovered in this study could have strong biological functions, and have potential as a serotype specific passive immunotherapeutic agent in the clinic for high-risk individuals. It is yet to be determined if these mAb will be able to protect in a therapeutic setting.

## Conclusions

In the current study, we report the isolation of several serotype specific anti-Pneumcooccal polysaccharide mAbs after PCV13 vaccination from a single subject. These results of dominant VH3 and JH4 germline gene usage with somatic hypermutation suggest a boosting of memory response as previously reported for the unconjugated polysaccharide vaccine. Interestingly, both the conjugated and the unconjugated Pneumococcal vaccines elicit similar antibodies from the pre-exisiting memory B-cells formed due to prior exposure to the vaccine antigens. Furthermore, the antibodies had potent in vitro and in vivo functional activities suggesting their active role in affording protection against Pneumococcal diseases.

## Additional files

Additional file 1: **Figure S1.** FACS sorting for single plasmablast cells. Freshly harvested peripheral blood mononuclear cells were stained with antibodies for flow cytometric detection of plasmablast cells. Plasmablasts were gated as shown, defined as lymphocytes/single cells/CD3-/CD19+ and low/CD27hiCD38hi/CD20 low, and sorted into single wells of a 96 well plate for RNA preservation and cloning. (PDF 1246 kb)

Additional file 2: **Figure S2.** Crystal structure and model of two antibody Fabs with VH3/JH4 gernline usage bound to polysaccharides epitopes. Crystal structure of mAb 023.102 bound to RGP (PDB: 4HIJ) [32], shown in cartoon (A) and surface view (B). The model of mAb 1A6 bound to ManNAc-FucNAc-GalNAc, shown in cartoon (C) and surface view (D). The model of 1A6 fab was built using 023.102 crystal structure as the template (PDB: 4HIJ) [32] by MOE v2018.0101 (Chemical Computing Group). The fragment of serotype 4 polysaccharide (ManNAc-FucNAc-GalNAc) [33, 34] was built in builder and dock with the CDRs of 1A6 fab model (MOE v2018.0101). Cyan, heavy chains; green, light chains; magenta, RGP; dark yellow, ManNAc-FucNAc-GalNAc. The figure was generated by PyMol 1.7.0.5 (Schrödinger). (PDF 2179 kb)

## Abbreviations

CDR: Complementarity-Determining Region
MOPA: Multiplexed OpsonoPhagocytic Assay
OPK: Opsonophagocytic Killing
PBMC: Peripheral Blood Mononuclear Cell
PCV13: 13-valent Pneumococcal Conjugate Vaccine
PPSV23: 23-valent Pneumococcal Polysaccharide Vaccine
