# Supplementary figures and images for: Human monoclonal antibodies isolated from a primary pneumococcal conjugate Vaccinee demonstrates the expansion of an antigen-driven Hypermutated memory B cell response
Source: BMC Infect Dis. 2018 Dec 4;18:613. doi: 10.1186/s12879-018-3517-7 (PMC6278343; doi:10.1186/s12879-018-3517-7)

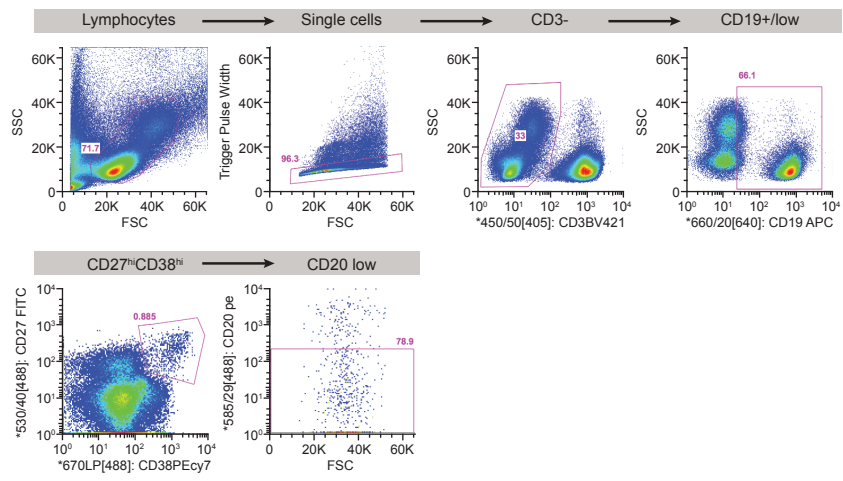

Supplement: Supplementary file 1 — Figure S1. FACS sorting for single plasmablast cells. Freshly harvested peripheral blood mononuclear cells were stained with antibodies for flow cytometric detection of plasmablast cells. Plasmablasts were gated as shown, defined as lymphocytes/single cells/CD3-/CD19+ and low/CD27hiCD38hi/CD20 low, and sorted into single wells of a 96 well plate for RNA preservation and cloning. (PDF 1246 kb) [file 12879_2018_3517_MOESM1_ESM.pdf]

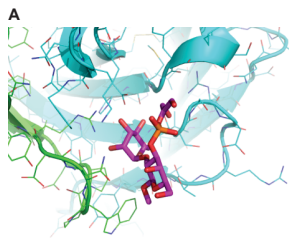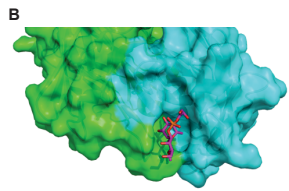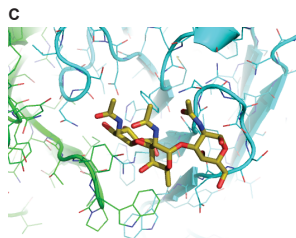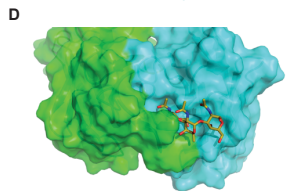

Supplement: Supplementary file 2 — Figure S2. Crystal structure and model of two antibody Fabs with VH3/JH4 gernline usage bound to polysaccharides epitopes. Crystal structure of mAb 023.102 bound to RGP (PDB: 4HIJ) [32], shown in cartoon (A) and surface view (B). The model of mAb 1A6 bound to ManNAc-FucNAc-GalNAc, shown in cartoon (C) and surface view (D). The model of 1A6 fab was built using 023.102 crystal structure as the template (PDB: 4HIJ) [32] by MOE v2018.0101 (Chemical Computing Group). The fragment of serotype 4 polysaccharide (ManNAc-FucNAc-GalNAc) [33, 34] was built in builder and dock with the CDRs of 1A6 fab model (MOE v2018.0101). Cyan, heavy chains; green, light chains; magenta, RGP; dark yellow, ManNAc-FucNAc-GalNAc. The figure was generated by PyMol 1.7.0.5 (Schrödinger). (PDF 2179 kb) [file 12879_2018_3517_MOESM2_ESM.pdf]
